# Hydrophobic and Metallophobic Surfaces: Highly Stable Non-wetting Inorganic Surfaces Based on Lanthanum Phosphate Nanorods

**DOI:** 10.1038/srep22732

**Published:** 2016-03-09

**Authors:** Sasidharan Sankar, Balagopal N. Nair, Takehiro Suzuki, Gopinathan M. Anilkumar, Moothetty Padmanabhan, Unnikrishnan Nair S. Hareesh, Krishna G. Warrier

**Affiliations:** 1 Materials Science & Technology Division, CSIR- NIIST, Thiruvananthapuram, India; 2 R&D Centre, Noritake Co. Ltd., Aichi, Japan; 3 Department of Chemistry, Amrita Vishwa Vidyapeetham University, Amritapuri, Kerala, India, 690525; 4 Materials Science & Technology Division, CSIR- NIIST, Thiruvananthapuram, India, 695019; 5 Academy of Scientific and Innovative Research (AcSIR), New Delhi, India

## Abstract

Metal oxides, in general, are known to exhibit significant wettability towards water molecules because of the high feasibility of synergetic hydrogen-bonding interactions possible at the solid-water interface. Here we show that the nano sized phosphates of rare earth materials (Rare Earth Phosphates, REPs), LaPO_4_ in particular, exhibit without any chemical modification, unique combination of intrinsic properties including remarkable hydrophobicity that could be retained even after exposure to extreme temperatures and harsh hydrothermal conditions. Transparent nanocoatings of LaPO_4_ as well as mixture of other REPs on glass surfaces are shown to display notable hydrophobicity with water contact angle (WCA) value of 120° while sintered and polished monoliths manifested WCA greater than 105°. Significantly, these materials in the form of coatings and monoliths also exhibit complete non-wettability and inertness towards molten metals like Ag, Zn, and Al well above their melting points. These properties, coupled with their excellent chemical and thermal stability, ease of processing, machinability and their versatile photo-physical and emission properties, render LaPO_4_ and other REP ceramics utility in diverse applications.

Hydrophobicity has a very crucial role in providing diverse functional properties to substrates and surfaces^1^,^2^,^3^,^4^,^5^,^6^. Though inorganic materials such as oxide ceramics are known to exhibit significant affinity towards water molecules, their surfaces can be made super hydrophobic either by micro-patterning or by surface treatments with organic modifiers^7^. Many plant leaves such as lotus, have patterned surfaces with hierarchical structures of micro and nanometer sizes. Several investigations in the past have reproduced similar patterns to create organic and inorganic hydrophobic surfaces leading to remarkable properties of water repellency and self-cleaning^8^,^9^,^10^. However, the instability of the organic/polymer coatings and the possible decay of the high surface area patterned surfaces in harsh environments make such ceramics unsuitable for challenging applications. Ceramic materials composing of metal and oxygen, in general, are known to exhibit significant affinity towards water because of the high feasibility of synergic hydrogen-bonding interactions possible between H_2_O molecules at the solid-H_2_O interface. In case of a common ceramic material like alumina (Al_2_O_3_), appreciable multi-mode H-bonding of its surface with H_2_O molecules get facilitated because of the electronic requirements of the atomic layer near the surface leading to complete wetting^11^,^12^.

An earlier work^1^,^13^, has highlighted the hydrophobic behavior of rare earth oxides (REOs, also known as lanthanide metal oxides, Ln_2_O_3_) and has also demonstrated their ‘super-hydrophobic’ nature when the sample surfaces were structured with ordered micro-roughness. The H_2_O repelling nature shown by REOs was explained based on one-way H-bond interactions manifested between H_2_O and Ln_2_O_3_ compared to the two-way and synergistic interactions that are often possible for H_2_O with other common wettable surfaces such as Al_2_O_3_. Such an argument induces the assumption that, the La^3+^ analogue La_2_O_3_ (which is the first member among 14 REOs) with an empty 4f orbital should also be an equally good candidate exhibiting such hydrophobic nature. However, La_2_O_3_ on exposure to atmosphere reacts readily with the moisture present in air leading to the formation of La(OH)_3_. As a matter of fact, the reaction with water in atmosphere leading to the formation of hydroxide seems to be common in other rare earth oxides as well^14^,^15^ irrespective of the presence of unfilled 4f orbitals in the rare earth element.

La being the cheapest and the most abundant among rare earth elements, it is important to develop a La-based chemically, thermally and mechanically stable material having easy processability and non-toxicity. Over the years, our group has been active in the development of LaPO_4_ based nanomaterials and structures for various functional applications that are unique to lanthanides^16^,^17^,^18^,^19^. In this paper, we have attempted to investigate in detail experimentally and also through first principles simulations, the surface wetting characteristics of LaPO_4_. We have shown that, LaPO_4_ could act as a highly stable substitute for its oxide counterpart in terms of its excellent hydrophobicity. The unique characteristic of non-wettability and non-reactivity towards molten metals may open up new frontiers in engineering applications.

## Experimental Procedures

LaPO_4_ nanoparticles for the disc fabrication and nanosol for the coating purposes were prepared by an aqueous sol-gel process as reported elsewhere^20^. The LaPO_4_ nano coatings were made on glass substrate using a dip coater supplied by KSV Instruments (Netherlands). The coated glass slides were dried at 50 °C for 5 h and then annealed at a temperature range 100–400 °C. The optical transparency was recorded using UV- Vis (Shimadzu, Japan) spectrophotometer. The contact angle values were obtained using Hamilton 500 microlitre needle with a drop volume of 3microlitre and flow rate of 1microlitre/sec. The surface roughness of the coatings was measured with atomic force microscopy (AFM). AFM images were obtained using an NTEGRA (NT-MDT) instrument operating in a tapping mode regime.

The LaPO_4_ nano powders were initially heated to 800 °C to avoid coarsening and 65wt% slurry of LaPO_4_ was prepared by ball milling for approximately 12 h after adding isopropyl alcohol (IPA) and 2wt% poly vinyl pyrrolidone (PVP). The slurry was dried, powdered and sieved to obtain powders, which were compacted uniaxially to form discs of approx. 50 mm diameter. The green samples of LaPO_4_ were sintered at 1400 °C/3 h (ramp rate of 3 °C/min) in a high temperature furnace and mirror polished to nullify errors in measurement. Phase identification of the discs was carried out using Philips PW 1710 X-ray Diffractometer using Cu Kα radiation in 2θ range. LaPO_4_ bars for finger tests with Aluminium molten metal were also prepared in similar way. The surface chemistry of LaPO_4_ pellet was analyzed using X-ray photoelectron spectroscopy (XPS) which was recorded on a XPS system (PHI 5000 Versa Probe II, ULVAC-PHI, INC., USA) using a monochromatic Al Kα X-ray source (1486.6 eV).

## Results and Discussion

### Hydrophobicity of Lanthanum Phosphate Nano Rods

**Figure 1:**
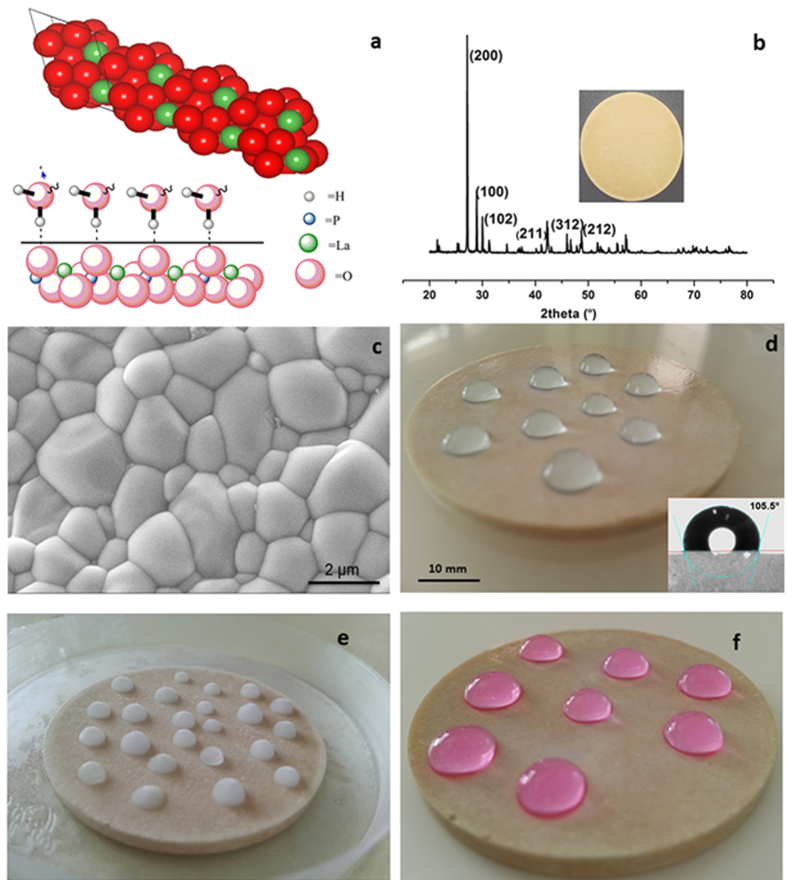
Hydrophobicity of LaPO_4_ established through (**a**) Model of LaPO_4_; structure and schematic representation of the nature of interactions between H_2_O and LaPO_4_ surface (**b**) XRD pattern for the monoclinic LaPO_4_ along with a photograph of the sintered disc sample (**c**) Polished and thermally etched microstructure of the LaPO_4_ disc sintered at 1400 °C (**d**) Water droplets sitting over the LaPO_4_ disc and the corresponding contact angle value (**e**) Frozen water droplets (**f**) Water droplets over a LaPO_4_ disc which was earlier hydrothermally treated in an autoclave at 200 °C for 24 h.

As mentioned previously, it is reported that La_2_O_3_ on exposure to atmosphere reacts readily with the moisture present in air leading to the formation of La(OH)_3_.We have attempted to investigate in some detail, the possible interactions of La_2_O_3_ as well as Nd_2_O_3_ with H_2_O and the results are presented in Supplementary Information S1. XRD and FTIR spectra shown in section S1 show clear match between La_2_O_3_ and La(OH)_3_ and convincingly prove that the rare earth oxide is susceptible to reaction with humidity in atmosphere leading to the formation of hydroxide. As a result of this interaction, the density of the material changes from 6.51 gcm^−3^ (La_2_O_3_) to 4.28 gcm^−3^ (La(OH)_3_), leading obviously to a volume expansion resulting in the collapse of La_2_O_3_ monoliths on continued exposure to atmosphere. Nd_2_O_3_ also follows a similar susceptibility to moisture exposure (S1) with time. The presented experimental data are in line with the previously reported results^14^,^15^. Given the fact that, the oxide of lanthanum is unstable and vulnerable to atmospheric moisture exposure we have explored the case of LaPO_4_ for possible hydrophobic properties. Additionally, the metal ions present in the phosphate analogue stay buried inside, more than its oxide counterpart. The bulky PO_4_^3-^ units surrounding the La^3+^ ions can make the metal ions much less available for the two-way synergic interaction involving La^3+^ and O of H_2_O molecules. The only way then H_2_O molecules can interact with LaPO_4_ surface is through H of water and O of the PO_4_^3-^ moieties (as shown in the schematic in Fig. 1a) in contrast to Al_2_O_3_-H_2_O where two-way synergic interactions lead to pronounced water wetting at the interface. Furthermore, we have carried out first principles simulations on LaPO_4_ (and some of the rare-earth oxides) in comparison with Al_2_O_3_ and were encouraged by the lower surface energy values of LaPO_4_ compared to those of La_2_O_3_, Gd_2_O_3_ and Al_2_O_3_, which also should aid in creating hydrophobic interactions as reported elsewhere^4^. For the surface energy calculations, the surface structure of the material was modeled based on slab approximation and first principles calculations were performed by the Projector Augmented Wave (PAW) method as implemented in Vienna Ab initio Simulation Package (VASP). Further details on the calculations are provided in S2 of Supplementary Information. The results showed that the surface energy of the [010] plane was only 0.77 J/m^2^ in the case of LaPO_4_, whereas the corresponding values were 0.92, 1.27 and 1.54 J/m^2^ for La_2_O_3_, Gd_2_O_3_ and Al_2_O_3_ respectively. Although the surface energy calculations were restricted to the [010] plane, the results obtained were very supportive of our experimental evidence on hydrophobicity and non-wettability to molten liquid metals discussed hereafter.

In order to test the hydrophobic properties under various conditions, we fabricated discs of LaPO_4_ and heat-treated them at a temperature of 1400 °C to realize fully dense shapes of monoclinic LaPO_4_ as shown in Fig. 1b. The discs were polished to minimize any effect of surface structure on further measurements. The surface characteristics of these discs were analyzed using XPS analysis (S3 of supplementary information). The microstructure obtained on polished and thermally etched surface of LaPO_4_ (SEM shown in Fig. 1c) is characteristic of a dense ceramic material. The hydrophobic nature of the material surface was evaluated using WCA measurements as shown in Fig. 1d. Employing sessile drop method and using a Hamilton 500 micro-liter needle with a drop volume of 3 μL and flow rate of 1 μL/sec (inset of Fig. 1d) we measured a contact angle value of 105.5° on LaPO_4_ monoliths. The water droplets were seen to move smoothly over the disc without any spreading.

### Stability under Extreme Conditions

Further, LaPO_4_ disc with water droplets kept in freezing condition (Fig. 1e) retained the shape (the droplets appeared as ice hemispheres over its surface) confirming that LaPO_4_ could also manifest its hydrophobicity well under sub-zero temperatures. The water (ice) droplets retained their shapes even after the disc was brought back to room temperature. Video images of the formation of ice hemi-spheres from colored water drops under a stream of liquid nitrogen had been captured (see S4 of Supplementary Information for video and image). This characteristic feature of LaPO_4_ should make the material attractive for developing icephobic^20^ and antifreeze coatings and thin films for a variety of energy efficient applications. To further evaluate the stability of the materials under harsh and extreme conditions, LaPO_4_ discs were subjected to severe hydrothermal treatment in a high-pressure autoclave. For this study, the discs were immersed in water and kept in the high-pressure autoclave at 200 °C for 24 h, thereby exposing them to a hydrothermal pressure of 1.55 MPa. The pellets retained their shapes without showing any signs of disintegration or weight loss even after this extreme hydrothermal treatment at very harsh conditions and the hydrophobicity was retained to the original level with contact angle values as before, 105.5° (Fig. 1f). The non-vulnerability and the excellent stability of LaPO_4_ materials in water even under such drastic conditions stand testimony to their extra-ordinary chemical and mechanical stability. Additionally, their suitability as a very reliable hydrophobic material and a stable inorganic coating for applications in pressure vessels and boilers is also evidenced by the results reported herein. For further confirmation of the chemical stability to corrosive environments, LaPO_4_ powders were subjected to treatment with strong mineral acids as well as alkalis (See supporting Information S5 for details). The XRD spectra of samples before and after the chemical treatment were found to match, demonstrating the corrosion resistance of the material. It is, hence evident that LaPO_4_ and its coatings could significantly enhance the durability of substrates against corrosion and therefore can replace successfully almost any conventional organo-silane coatings. LaPO_4_ thus emerges as a very stable, inexpensive and versatile substitute for the highly hygroscopic oxide counterpart for achieving hydrophobicity in combination with its remarkable thermal and chemical stability.

### LaPO_4_ Hydrophobic Nano Coatings

**Figure 2:**
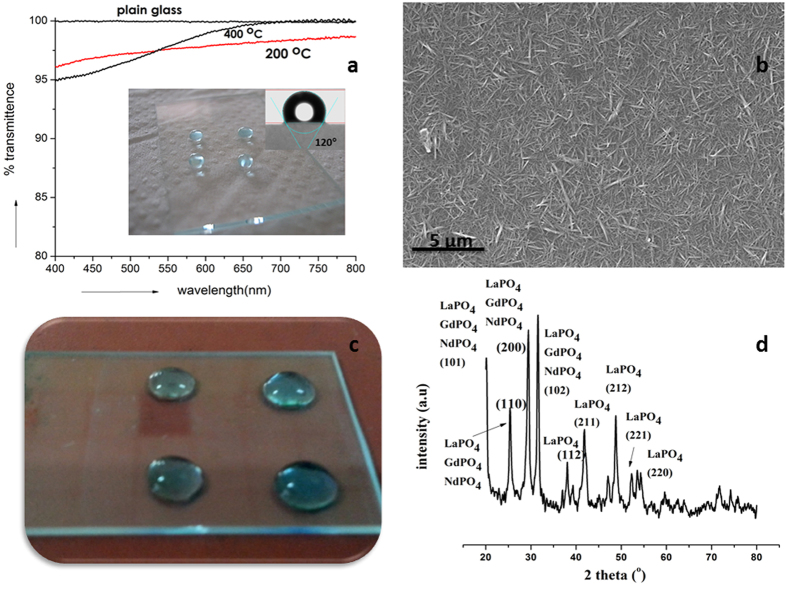
Hydrophobicity achieved for LaPO_4_ coated thin film (**a**) Water droplets sitting over LaPO_4_ coated glass plate and the high optical transparency recorded for the films (**b**) SEM image showing spike-like arrangement of LaPO_4_ nanorods over the glass surface (**c**) Mixed REP sol coated glass slide showing hydrophobic character (**d**) Diffraction pattern obtained for mixed rare earth phosphates.

To further illustrate the importance of the material in real life applications, we have made nanocoatings of lanthanum phosphate by dip-coating glass plates with LaPO_4_ nanosol prepared as reported elsewhere^21^. The coatings obtained after heat treatment in the temperature range of 200–400 °C had an average thickness of ~200 nm as estimated using SEM (See Supplementary Information, S6). The coated glass substrates showed high optical transparency (>95%), as confirmed by UV-Vis spectral measurements presented in Fig. 2a, very much desirable from application point of view. Sessile drop measurements of the LaPO_4_-coated glass plates showed very high WCA of 120° (Fig. 2a) when compared to the value of 14° measured with the uncoated glass plate (See S7 of Supplementary Information). Water droplets attained near-spherical shapes over the coated glass surface and the higher WCA values observed in these cases could be attributed mainly to the intrinsic hydrophobic nature of the material and partly to the unique morphology of the film surface obtained by the random arrangement of LaPO_4_ nanorods (Fig. 2b). Atomic force microscopic (AFM) analysis (See S8 of Supplementary Information) provides the surface structure of the coating. The uncoated glass surface had a roughness of ~7 nm while the coated surface showed roughness of ~ 34 nm due to contribution from the rod-like morphology of the LaPO_4_ particles as well as their assembly. The rods appeared to have organized to form a spike like arrangement over the surface forming a rougher surface. The morphology of the coated surface is complimentary to the benefits obtained by virtue of the restricted H-bond interactions as mentioned earlier and should have contributed partly to the very high water contact angle observed for the coated sample^22^.

Since most of the other rare-earth phosphates also have almost similar structural features as LaPO_4_ we have attempted studies with a few other REPs, anticipating properties similar to LaPO_4_. Mixed rare earth phosphate sol (containing LaPO_4_, GdPO_4_ and NdPO_4_) synthesized using similar procedure was coated over glass plates and the coated surface obtained showed almost similar hydrophobic behavior (Fig. 2c) as in the case of LaPO_4_ indicating the possibility of using all kinds of mixtures and combinations of REPs for diverse functional applications. The structural similarity of various REPs as evidenced from the XRD patterns for the dried sol containing mixture of several rare earth phosphates (Fig. 2d) and the hydrophobicity seen for the REP-mixture coated surface (Fig. 2c) opens up possibilities for fabricating very stable ceramic hydrophobic surfaces from cheap rare earth resources containing all types of mixture of rare earth metal ions. Moreover, LaPO_4_ could also be made in phase pure form by the controlled addition of phosphoric acid to a variety of lanthanum salts such as nitrates, carbonates and chlorides^16^,^17^,^18^,^19^. This indicates the possibility of generating versatile hydrophobic materials easily and economically from any rare earth based chemical precursors or even ‘rare-earth wastes’ that could otherwise cause serious waste disposal concerns.

Currently rare-earth oxides are being widely used as biological sensors/probes and for bio-assays in diagnostics, but the health hazards associated with these oxides have been of much concern. It is known that REOs can easily strip phosphate moieties from lipid bilayers in biological systems^23^, leading to the formation of very stable and inert phosphate phases. This phosphate abstraction can cause severe cellular and pulmonary damage to the living systems leading to lung fibrosis and therefore substitution of REOs by the chemically and biologically inert LaPO_4_ and other REPs could be a viable strategy for safe biological use^24^,^25^.

### LaPO_4_ as Metallophobic Surface

**Figure 3:**
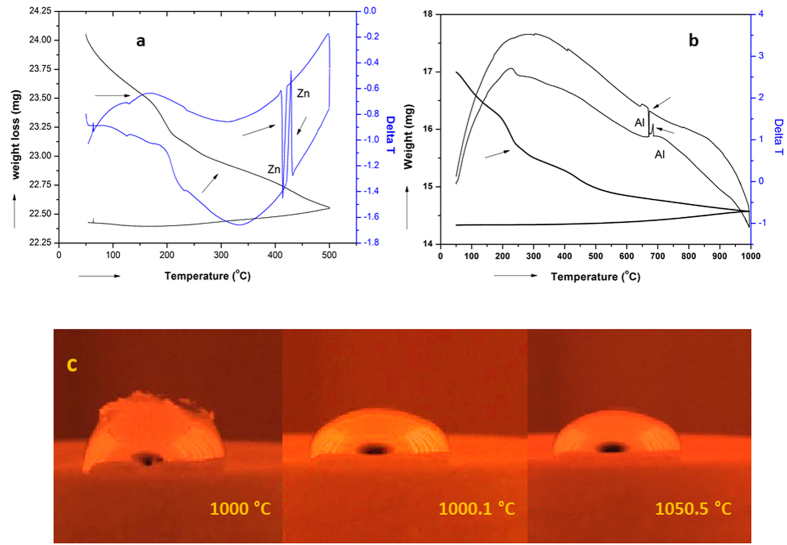
Non-Reactivity of LaPO_4_ with molten metals (**a**) TG-DTA patterns of the powder mixture containing LaPO_4_and Zn (**b**) TG-DTA patterns of the powder mixture containing LaPO_4_ and Al (**c**) Images of molten Ag metal placed over a LaPO_4_ pellet at temperatures close to and above melting temperature of Ag.

Yet another unique characteristic of LaPO_4_ which we demonstrate in this work is its excellent non-wetting/non-reactive nature to molten liquid metals even well above 1000 °C. TGA studies on powder mixtures of LaPO_4_ with Al and Zn metal powders in separate experiments clearly showed peaks corresponding to melting of the metal constituent on heating and solidification of melt on cooling (Fig. 3a,b). These experiments conclusively prove that the metal particles or the melt did not react with LaPO_4_ even while melting. In another experiment, a pellet of LaPO_4_ immersed in molten Zn, obtained by heating the pellet over Zn flakes, could be easily taken out and the impression of the pellet could be seen clearly on the solidified Zn metal (See 9 of Supplementary Information).The clean interface between the metal and the ceramic confirmed the non-reactivity and non-wettability of the molten Zn metal to LaPO_4_. This was further confirmed by analyzing the EDAX of the LaPO_4_ pellet surface, wherein no trace of Zn element was detected over the surface of the pellet (See S10 of Supplementary Information). The durability of the sample against corrosion and reactivity to molten Zn metal were studied at 500 °C/8 h using thermal analysis, the results of which convincingly showed the excellent non-reactivity of LaPO_4_ even on prolonged exposure (See S9, image c of Supplementary Information).To further test the long-term durability of the substrate against molten metals, zinc granules were kept on a lanthanum phosphate disc for 8 h at 500 °C. The substrate has shown no signs of any reactivity or surface damage after the exposure. We have also dipped sintered bars of LaPO_4_ in molten Al to test its non-wettability to the Al melt. The bars could be retrieved without any adherence of the metal on its surface confirming the non-reactive and non-wetting nature of LaPO_4_ (See S11 of Supplementary Information) to the molten metal. In yet another experiment, we have imaged a droplet formed by molten Ag over LaPO_4_ surface (Fig. 3c). The captured images just before complete melting and during different time intervals after melting in the furnace maintained at ~1000 °C and ~1050 °C are shown in the Fig. 3c. The large contact angles of the melt on the LaPO_4_ surface are clearly evident from the figures. The substrate sample could be retrieved with no surface damages due to reactivity or wetting with the molten silver metal. The excellent non-wettability to molten metals should allow the use of LaPO_4_ in various engineering applications.

## Conclusions

In summary, we have demonstrated that LaPO_4_, possesses remarkable intrinsic hydrophobicity both at ambient and sub-zero temperatures. The excellent non-wetting and non-reactive abilities of LaPO_4_ towards molten metals even above 1000 °C is also validated by way of high temperature contact angle measurements. LaPO_4_ in the form of thin layer coatings on glass surfaces showed excellent hydrophobicity with WCA as high as 120°. The inherent hydrophobic and non-wetting characteristics of lanthanum phosphate in combination with its excellent chemical stability thus offer opportunities to apply this inexpensive ceramic material for applications such as anti-icing coatings, anti- corrosive coatings as well as anti- fouling coatings. In addition, the excellent machinability of this material should help in the fabrication of lab on chip devices^26^,^27^, for running even harsh chemical reactions. Rare earth phosphates in general and lanthanum phosphate in particular thus emerge as a material which could be harnessed for diverse functional applications.

## Additional Information

**How to cite this article**: Sankar, S. *et al.* Hydrophobic and Metallophobic Surfaces: Highly Stable Non-wetting Inorganic Surfaces Based on Lanthanum Phosphate Nanorods. *Sci. Rep.* **6**, 22732; doi: 10.1038/srep22732 (2016).

## Supplementary Material

Supplementary Information

Supplementary Video
