# Supplementary material for: Hydrophobic and Metallophobic Surfaces: Highly Stable Non-wetting Inorganic Surfaces Based on Lanthanum Phosphate Nanorods
Source: Sci Rep. 2016 Mar 9;6:22732. doi: 10.1038/srep22732 (PMC4783694; doi:10.1038/srep22732)
Supplement: Supplementary Information [file srep22732-s1.pdf]

Supplementary Information

Hydrophobic and Metallophobic Surfaces: Highly Stable Non-wetting Inorganic Surfaces Based on Lanthanum Phosphate Nanorods

Sasidharan Sankar, Balagopal N. Nair, Takehiro Suzuki, Gopinathan M. Anilkumar, Moothetty Padmanabhan, Unnikrishnan Nair S. Hareesh* and Krishna G. Warrier*
*Email: hareesh@niist.res.in, warrier@niist.res.in

S1) Change in physical and chemical structure of La_2O_3 & Nd_2O_3 on atmospheric exposure

We attempted toinvestigate in some detail, the possible interactions of La_2O_3 and H_2O and the presented experimental data are in line with the previously reported results (References13, 14 of the manuscript). For examining the behaviour of La_2O_3, we made compacted pellets from its powder (Alfa Easer, 99.9 %) and sintered them at 1400 ºC in air atmosphere. The samples were taken out at ~300 °C while cooling from the sintering temperature. The weight increase and shape change of the compacted pellets under atmospheric conditions were tracked and recorded systematically over time and the resulting changes are represented in Figure 1a (top part). The sample weight increased gradually and was accompanied by a dramatic increase in the volume of the pellet leading to its fracture and complete disintegration within 24 h. Through XRD, TGA, FTIR and chemical analysis we could establish that La_2O_3 on exposure to atmosphere reacts readily with the moisture present in air leading to the formation of La $(OH)_3$ (Figure 1b). The TGA and PXRD patterns of the final product are shown in Figure 1(c-d). The TG data of the resulting powdery product presented in Figure 1c indicate weight losses in two distinctive temperature regimes of 300-400 °C and 450-550 °C which finally yielded the original La_2O_3. The PXRD pattern of the air-exposed product (Figure 1d) showed peaks that could be well indexed to $La(OH)_3$. The present experimental data are in line with published results (Reference 13 &14 of the manuscript) and clearly indicate the considerable affinity of La_2O_3 even to atmospheric H_2O. As a result of this reaction, the density of the system also changed from 6.51 gcm^{-3} (La_2O_3) to 4.28 gcm^{-3} ($La(OH)_3$), leading obviously to a volume expansion resulting in the collapse of La_2O_3 monolith on continued exposure to atmosphere as schematically shown in Figure 1a. We have observed that Nd_2O_3 is also air-sensitive and showed a similar reaction in atmospheric condition (Figure 2) although the kinetics of the reaction was much slower than in the case of La_2O_3. Though less pronounced most of the other REOs were also reported to be susceptible to atmospheric moisture (Reference 13 &14 of the manuscript).

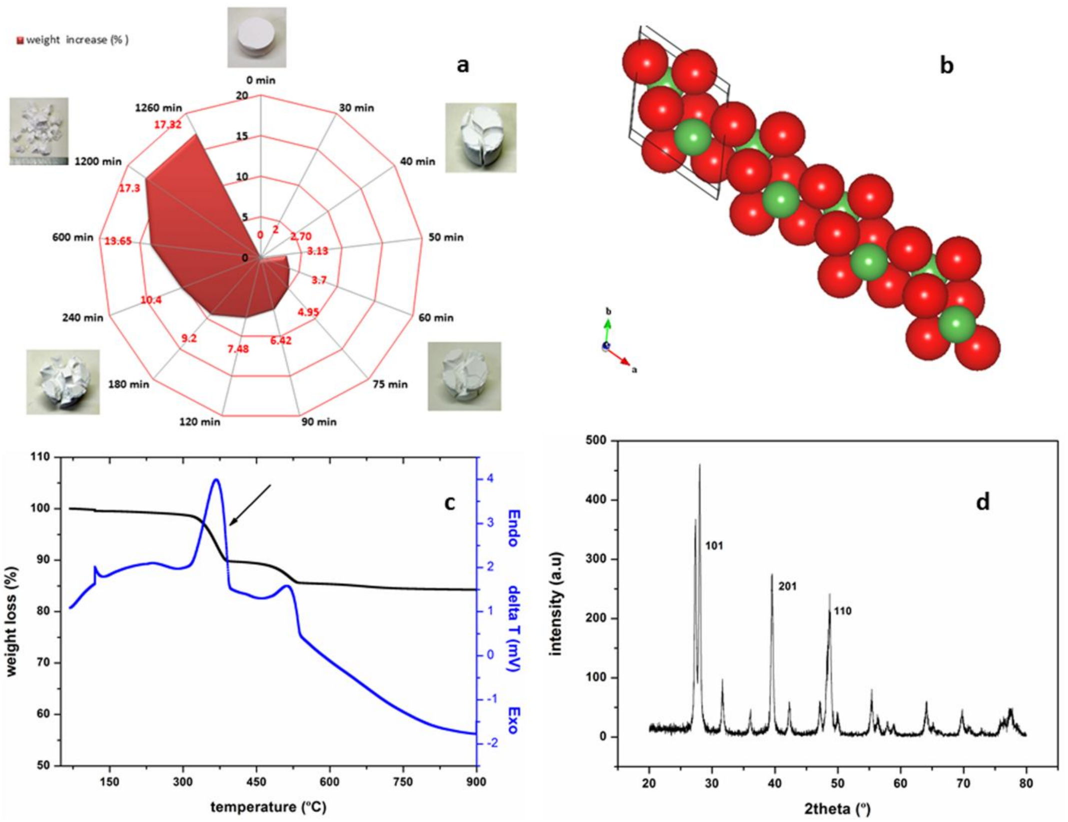

Figure 1 Chemical and physical changes of La_2O_3 on atmospheric exposure**(a**) Graphical representation of increase in weight with time of the air-exposed powder compact along with its photographs taken at regular intervals (**b**) The $La(OH)_3$ structure (**c**) TG and DTA traces of the final powdery material confirming the de-hydroxylation steps of $La(OH)_3$ yielding La_2O_3 on heating (**d**) The PXRD pattern obtained after atmospheric exposure which could be indexed to $La(OH)_3$ structure (JCPDS File No:36-1481).

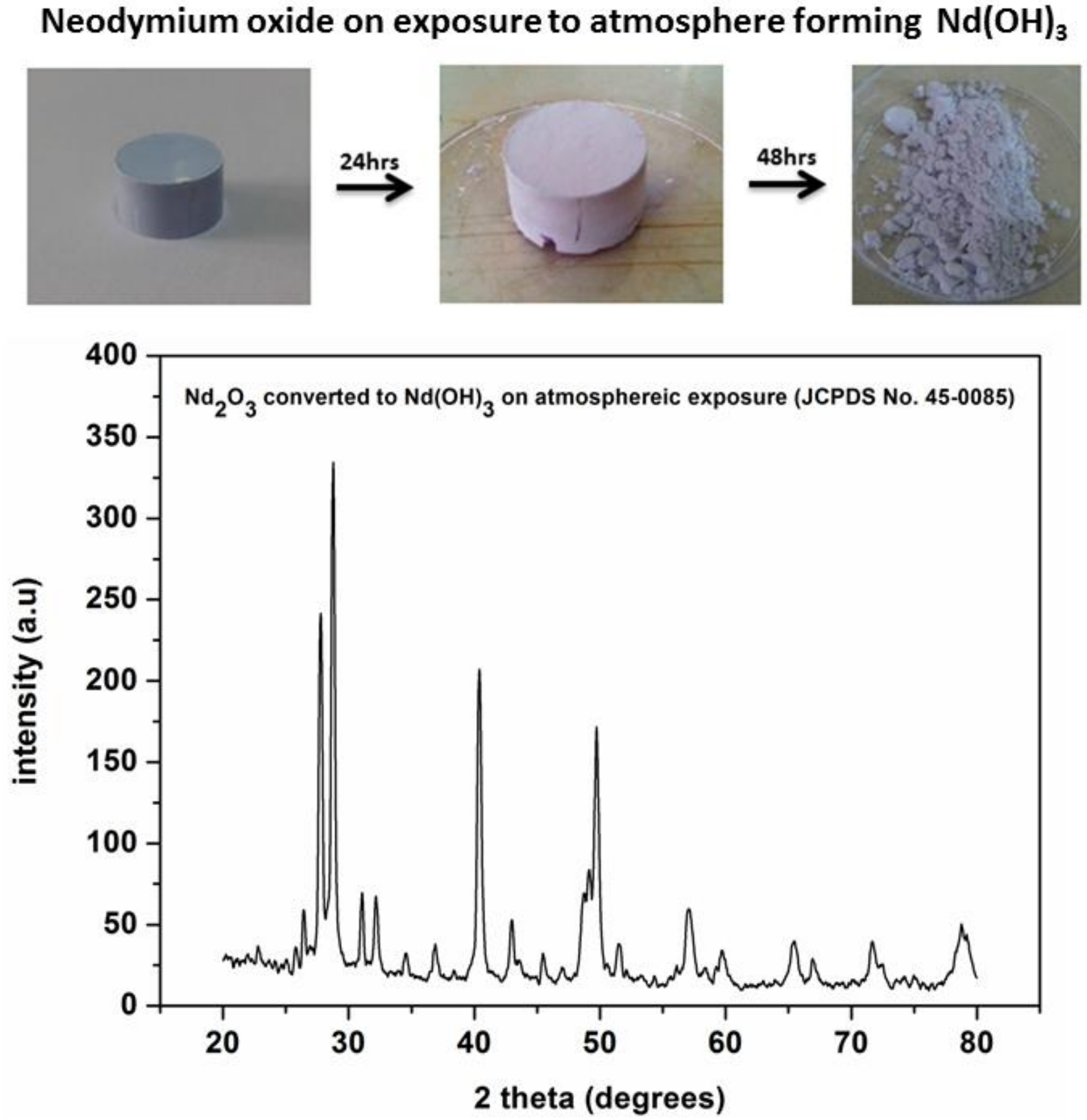

Figure 2 Disintegration of Nd_2O_3 pellet with time on air exposure and the corresponding XRD pattern showing peaks corresponding to the hydroxide phase Nd $(OH)_3$.

FT-IR Analysis

We have recorded FT-IR spectra of our $LaPO_4$ samples to confirm their chemical identity and purity and also the spectra of La_2O_3 and $La(OH)_3$ to explain some anomalous observations we have found in the case of some of the rare-earth oxides which were projected recently (Reference 1 of the manuscript) for their super hydrophobicity. Given below are the FT-IR spectra of these three compounds (figure 3, S1). In the case of $LaPO_4$ we get absorption peaks at 1067, 986, 947, 923, 632, 608, 540 and 500 cm^{-1}. The spectra could be easily interpreted knowing the T_d symmetry of PO_4^{3-} unit present in $LaPO_4$ and also based on their reported values (Reference 8 of the manuscript). Among these observed absorptions the split doublet seen at 1067 and 986 cm^{-1} are typical of ν_3 mode of P-O asymmetric stretching vibration while the finely split peak around 947 cm^{-1} is the characteristic ν_1 mode of P-O (symmetric stretching) vibration peak of $LaPO_4$. Similarly the split triplet seen at 632, 608 and 540 cm^{-1} in the spectrum are very characteristic of ν_4 mode of O-P-O (asymmetric bending) and the absorption around 500 cm^{-1} the expected ν_2 mode of the O-P-O (symmetric bending) vibration of monoclinic form of $LaPO_4$. All these values confirm the structural identity of our $LaPO_4$ samples. The results are in accordance to our experimental studies on the atmospheric reaction of lanthanum oxide leading to the formation of $La(OH)_3$, (Figure 1- S1).The peak obtained at ~3612 cm^{-1} indicates the stretching mode of O-H in La $(OH)_3$ as well as the oxide. The peak at 1440 cm^{-1} for the atmospheric exposed La $(OH)_3$ indicates absorption of CO_3^{2-} on to the surface.[1]

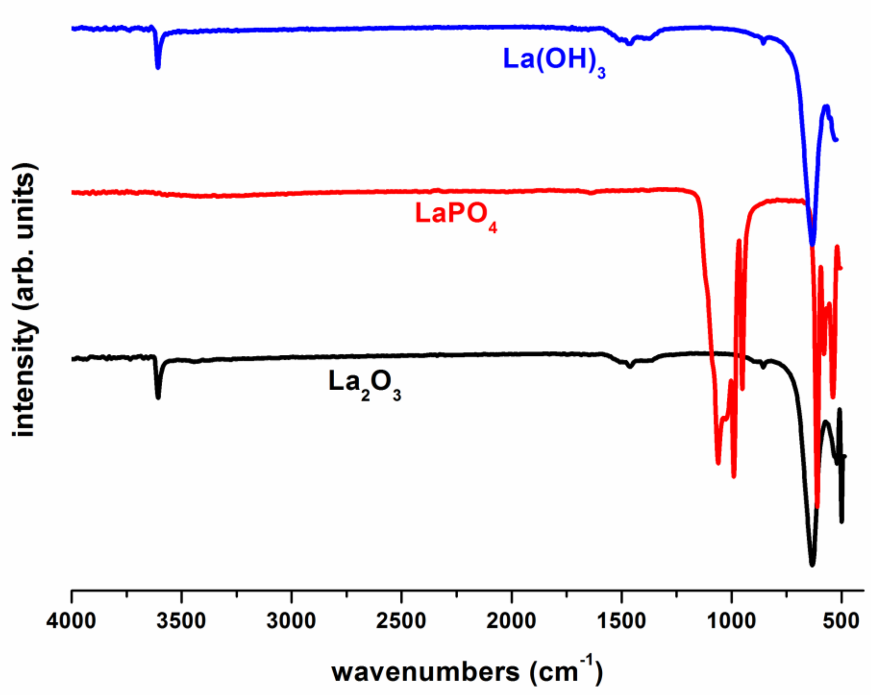

Figure 3 FT-IR spectrum of atmospherics exposed La_2O_3, $LaPO_4$ and $La(OH)_3$

S2) Surface energy calculations

We have calculated surface energy of lanthanum phosphate ($LaPO_4$) and compared it againstthe surface energy values of La_2O_3, Gd_2O_3 and Al_2O_3. For calculation of surface energy we have adapted the following method

Surface structure of $LaPO_4$ was modelled using slab approximation. Surface slab models were constructed based on corresponding optimized bulk crystal. A 2 × 2 cell was used for (010) $LaPO_4$ surface. The slabs were constituted of sixteen stoichiometric layers. The slabs were sandwiched by vacuum layers with a thickness of at least 10 Å (see Figure 4, S2).

Surface energy, γ_{lmn} was obtained using the difference in the Gibbs free energies of surface and that of corresponding bulk for unit surface area.

Gibbs free energy G is given by

$$G = U + pV - TS,$$

where U is the internal energy of the system, p is the pressure and S is the entropy.

The difference in Gibbs free energies between the surface model and the bulk model of solid was calculated by the above method. We ignored zero-point energy and pV term as well as TS term because their differences in surface model and bulk model could be much smaller than the difference of internal energy. U is approximated by the total energy of the system. Therefore, Gibbs energy difference of the system becomes equal to the difference of the total energy, E^{total}.

Hence, surface energy could be obtained using the difference in the total energy of slab model and that of corresponding bulk model per surface area.

$$\gamma_{lmn} = \frac{E_{\mathrm{slab}}^{\mathrm{total}} - nE_{\mathrm{bulk}}^{\mathrm{total}}}{2A}$$

First principles calculations in this paper were performed by the PAW method as implemented in VASP code. [2-4] The generalized gradient approximation with the exchange-correlation functional proposed by PBE[5] was employed together with GGA+U approach [6, 7] in the simplified spherically averaged version. U_{eff} = 7.5 eV is applied to the La 4f states to correct their position relative to La 5d levels. The plane-wave cutoff energy was 400 eV.

As a first step, the cell parameters of bulk model were calculated. For this, the *k*-point sampling condition ensured a good accuracy of total energies for each crystalline species within 1 meV/atom. All atomic positions and cell parameters were allowed to relax until their forces converged to be less than 0.02 eV/A to obtain structure for bulk.

Then, structural relaxation of slab model constructed based on optimized bulk model was performed only on the position of atoms.

.

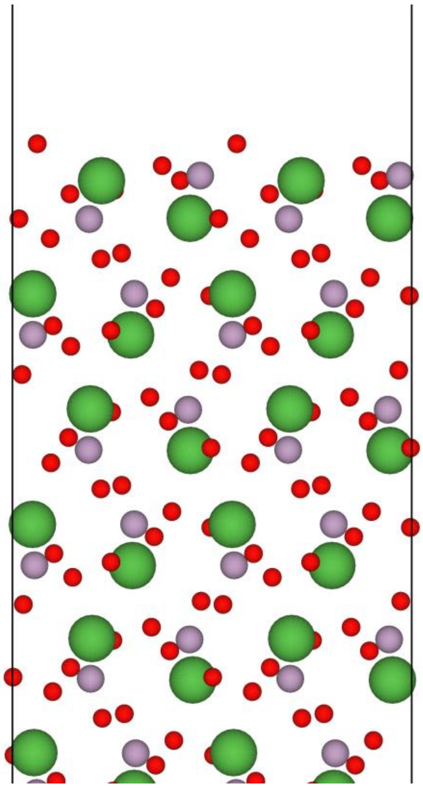

Figure 4 Surface model picture of $LaPO_4$ (La: Green, O: Red, P: Purple)

Calculated geometric parameters for bulk $LaPO_4$ crystals (monoclinic) compared with experimental data are shown in table below. As shown, calculated lattice parameters are in good agreement with corresponding experimental data. Similar calculations were performed for Gd_2O_3 (monoclinic), La_2O_3 (monoclinic) and Al_2O_3 (α) crystal structures as well. The surface energy values calculated for the four structures are reported in the paper.

	Exp. data [8]	(This work) calc.	Error [%]
a [Ang.]	6.482	6.51	0.4
b [Ang.]	7.057	7.09	0.4
c [Ang.]	8.2691	8.30	0.4
β [deg.]	126.5	126.7	
La-O	2.5	2.5	
La-P	3.282	3.30	
P-O	1.524	1.54	
O-O	2.427	2.44	

Table 1.Calculated geometric parameters of bulk $LaPO_4$ crystals

S3) Surface Characteristics of $LaPO_4$ by XPS

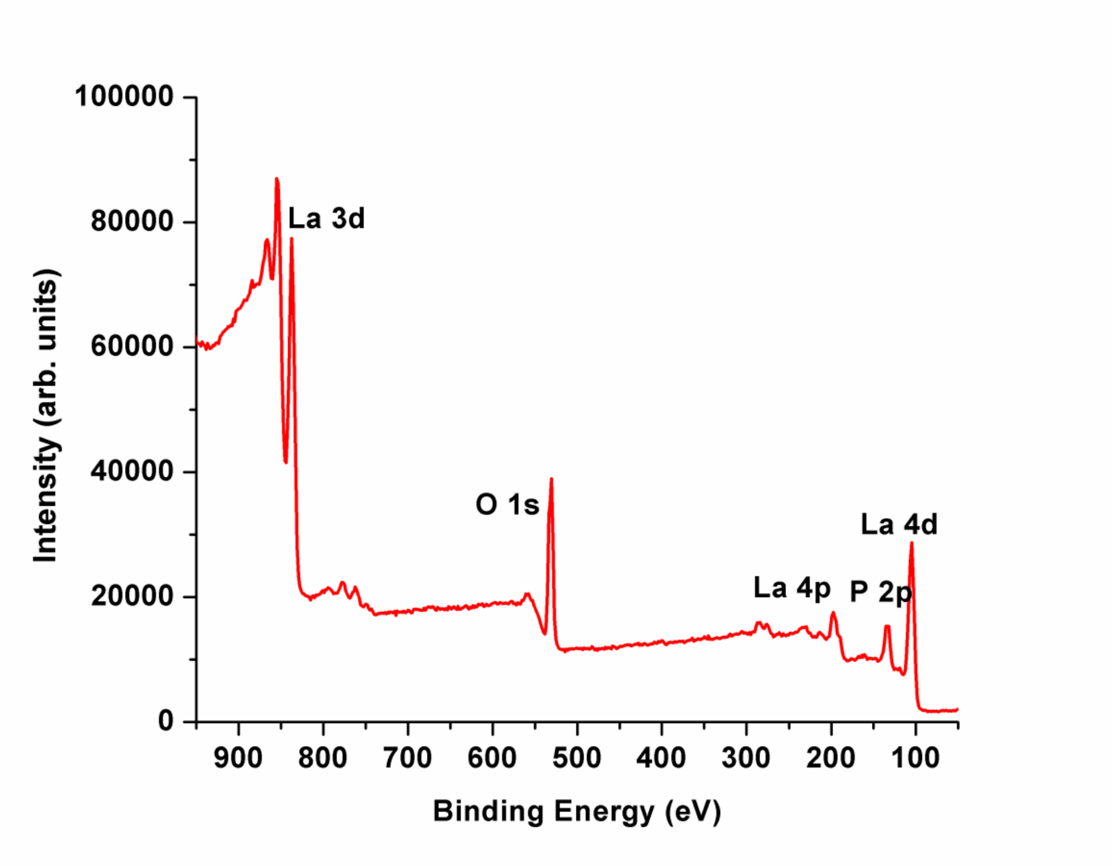

XPS analysis of the sintered $LaPO_4$ pellet was carried out to analyze the surface characteristics and phase purity of the samples. The spectrum was taken by an online eclipse data system (UK). The $LaPO_4$ powder was compacted to 8mm diameter and placed on the sample holder. The sample was stored in chamber with vacuum of 10^{-9} – 10^{-10} Torr (1 torr = 133.3 Pa). The sample was then transferred to the analysis chamber for the acquisition of the data with the following steps; 0.2 eV, 250 ms dwell time, 0.7 eV resolution and 10 scans. The binding energy values were determined with respect to the carbon (C 1s) line at 284.6 eV which is present due to unexpected reasons. The standard deviation corresponding to the peak position was found to be 0.5 eV. We could clearly see the highly characteristic peaks due to La^{3+} (3d), La^{3+} (4p) and La^{3+} (4d), 2p of P and also of 1s of O present in $LaPO_4$ (which are marked in the spectrum). Based on the analysis the sample surface is found to be having pure form of the expected $LaPO_4$ phase, devoid of much contamination except negligible % of C.

The observed binding energies from the surface analysis are La 3d (852.2 eV), O 1s (532 eV), P 2p (133 eV).

S4) Ice formation from coloured water droplets in a stream of liquid Nitrogen

(*Video (S4) attached separately***)**

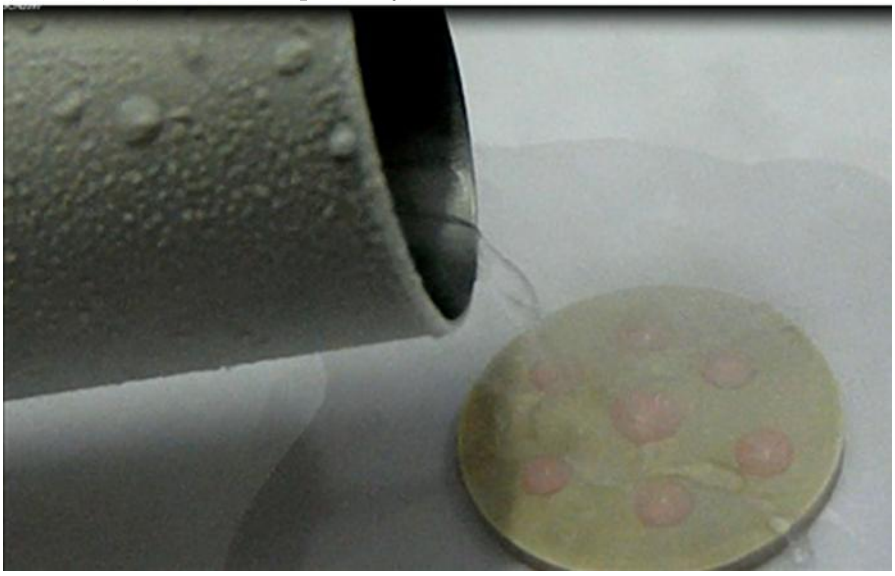

The above image shows when the water droplets over surface coming contact with liquid nitrogen freeze over time. The droplets after reaching room temperature retain the shape showing the stability of the phosphate.

S5) Lanthanum Phosphate stability in chemical environments

In order to ascertain the stability and inertness of $LaPO_4$ for wide applications in diverse chemical environments, we checked the reactivity of $LaPO_4$ samples with various strong acids and bases. In a typical reaction a suspension of about 3 g of $LaPO_4$ powder was heated with conc. H_2SO_4 for more about 2h and after cooling the reaction vessel the powder was filtered, dried and analysed using powder X-ray diffraction. Similar reactions were carried out on fresh samples of $LaPO_4$ using other acids like conc. HNO_3 and strong alkali like NaOH and the powder XRD were taken in all the cases to check their chemical identity and purity. In all the cases we found that even in powder form $LaPO_4$ was never reacting with

strong acids and bases even in extreme conditions and the samples remain completely inert retaining 100% phase purity. The PXRD peaks of the treated samples were matching in all the cases to those of pure $LaPO_4$. The dried samples obtained after the reactions also showed quantitative retrieval which further indicate the complete inertness of the material in those harsh chemical environments.

.

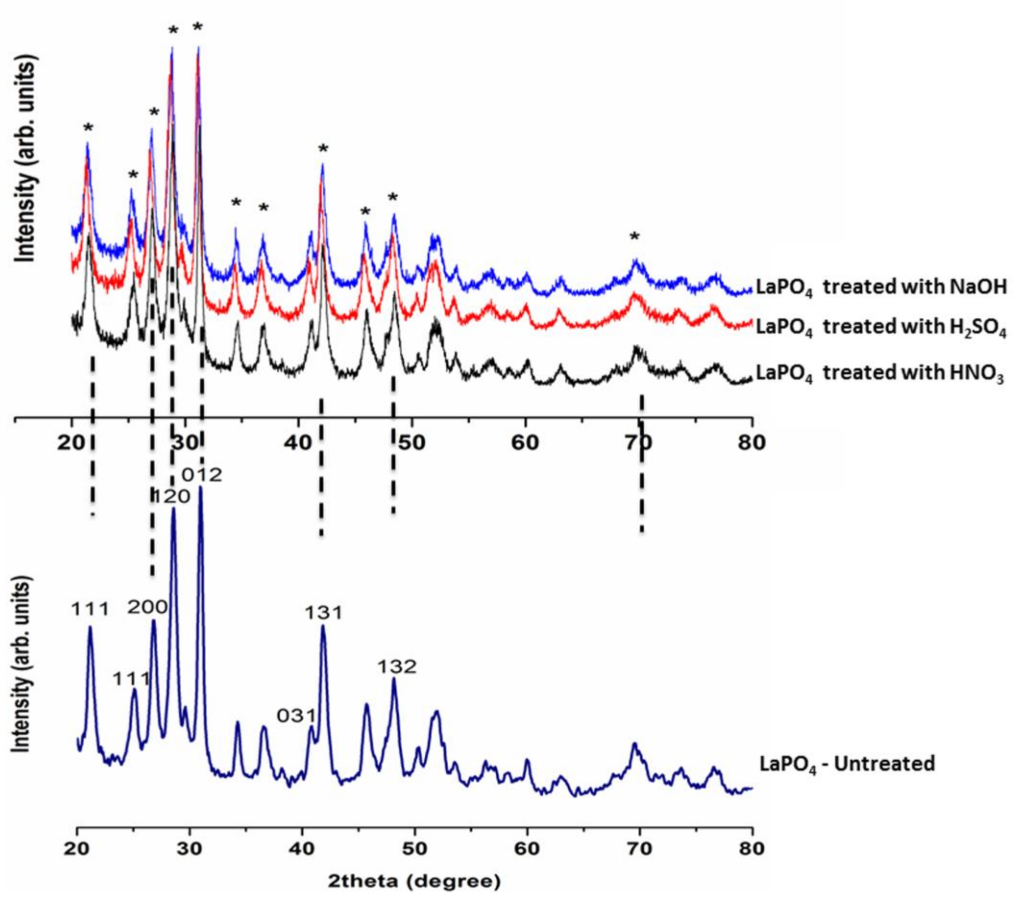

S6) Thickness of the thin $LaPO_4$ film obtained

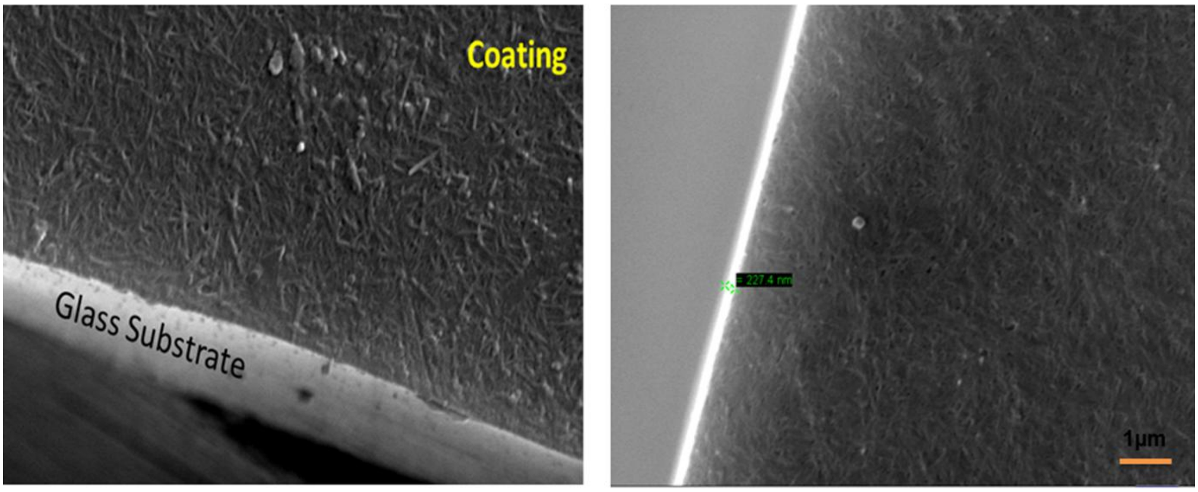

The SEM micrograph obtained for the $LaPO_4$ on glass surface shows the rod morphology of the phosphate and gives the average thickness of the coating as ~ 220 nm

S7) WCA measurement on uncoated and coated glass plates

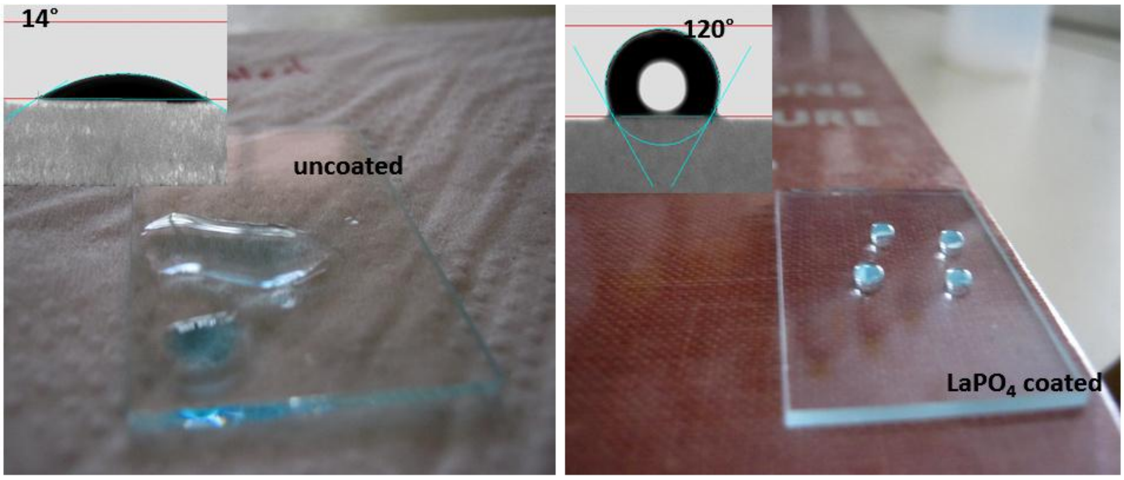

The photographs show the wetting of the uncoated glass surface and $LaPO_4$ coated glass surface by water. Water spreads over the uncoated surface due to the very low WCA of 14° (inset) when compared to the LaP coated hydrophobic glass surface with 120° WCA. The transparency of the coating is also very much evident from the photographs.

S8) Atomic force microscopic images and roughness measurement

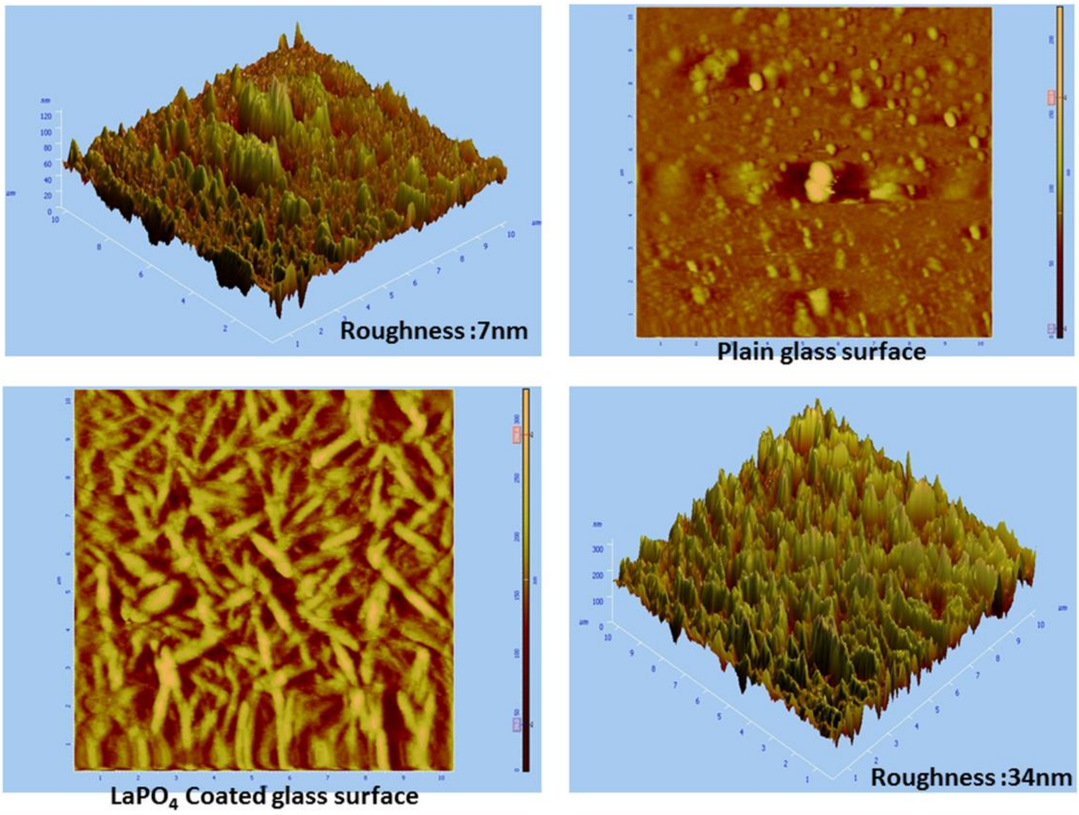

The uncoated plain glass surface has a very low surface roughness as per the AFM measurements carried out. The uncoated glass surface had a roughness of ~7nm while thecoated surface showed increased roughness of ~ 34nm.

S9) $LaPO_4$ reactivity with Zn metal

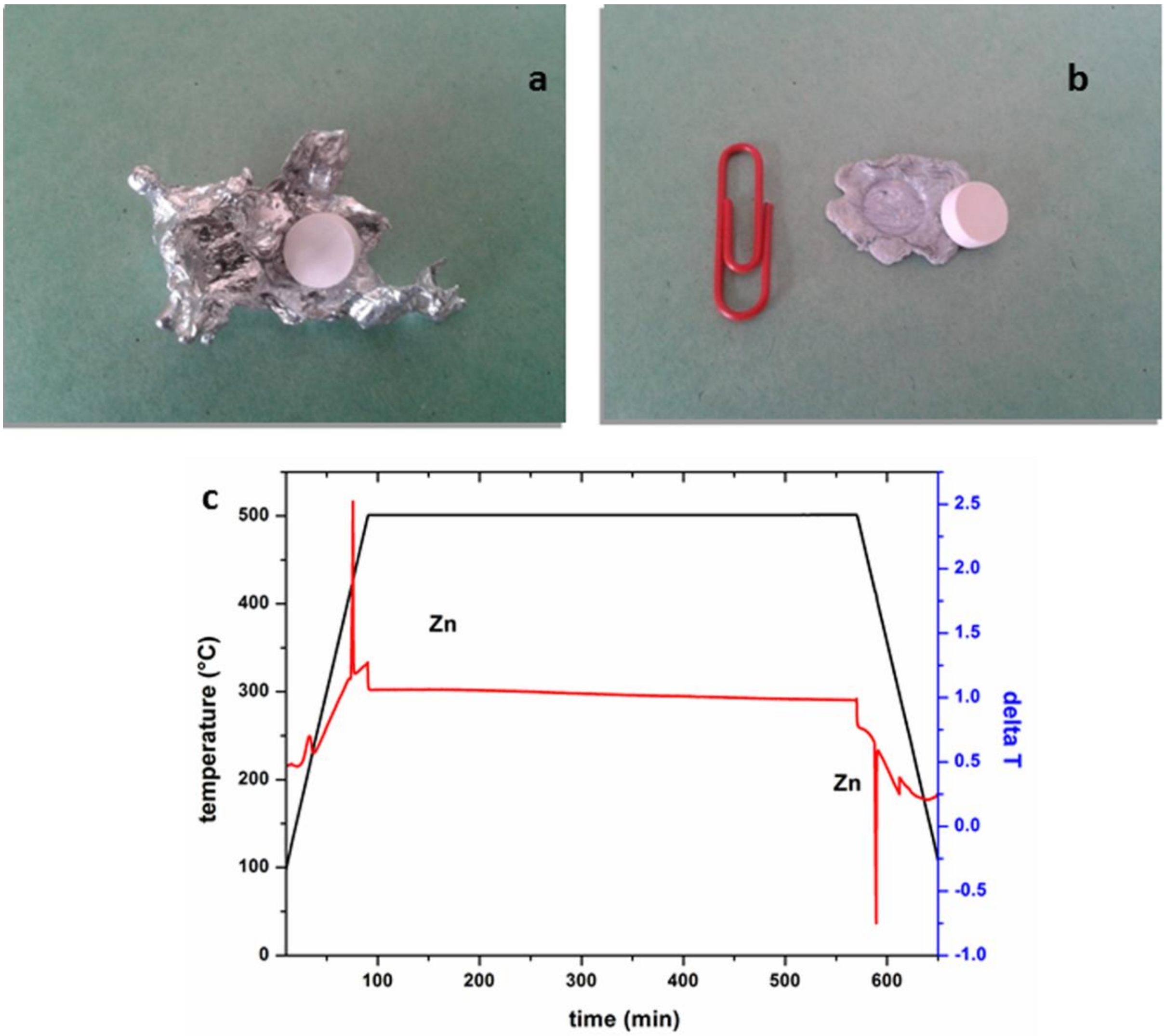

Lanthanum phosphate pellet wrapped in flakes of Zn metal (**a**) was heated above its melting point. After cooling we could see an impression of pellet on the surface of the melted Zn surface (**b**) and $LaPO_4$ didn't show any reaction with the metal which was further confirmed by taking EDAX of the pellet surface under study. No peak of Zn was obtained during the analysis (see S10).

Inorder to test the durability of the material under long term exposure to molten metals, Zn metal flakes mixed with $LaPO_4$ nanoparticles were kept under N_2 flow for 8h at 500 °C in a TG-DTA analyser.The DTA thermogram as in (**c**) below showed peaks corresponding tomelting and solidificationin the same temperature range evidencing absence of any reaction between the ceramic and metal during the long term exposure.

S10) EDAX Spectrum for the $LaPO_4$ pellet heated with Zn metal flakes.

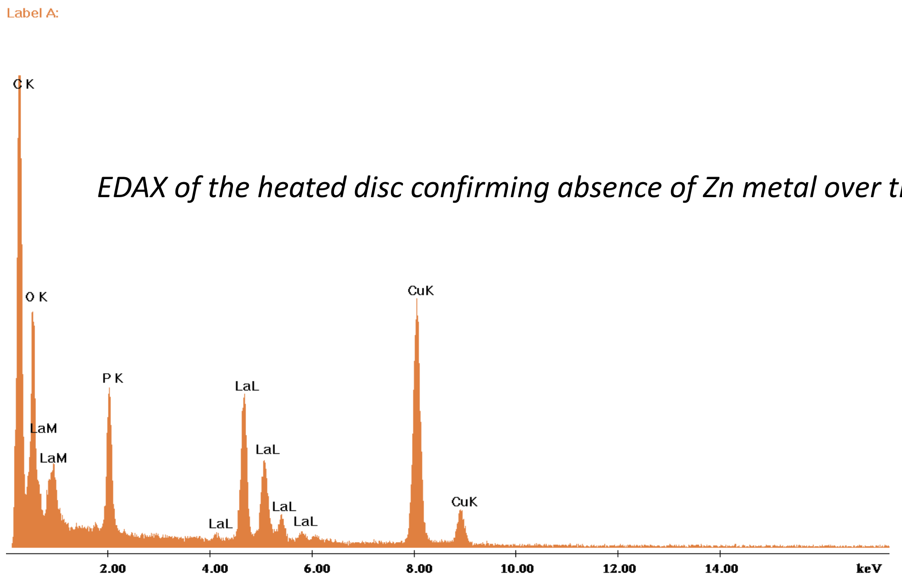

EDAX of the heated disc confirming absence of Zn metal over the surface

S11) $LaPO_4$ reactivity study with molten aluminum

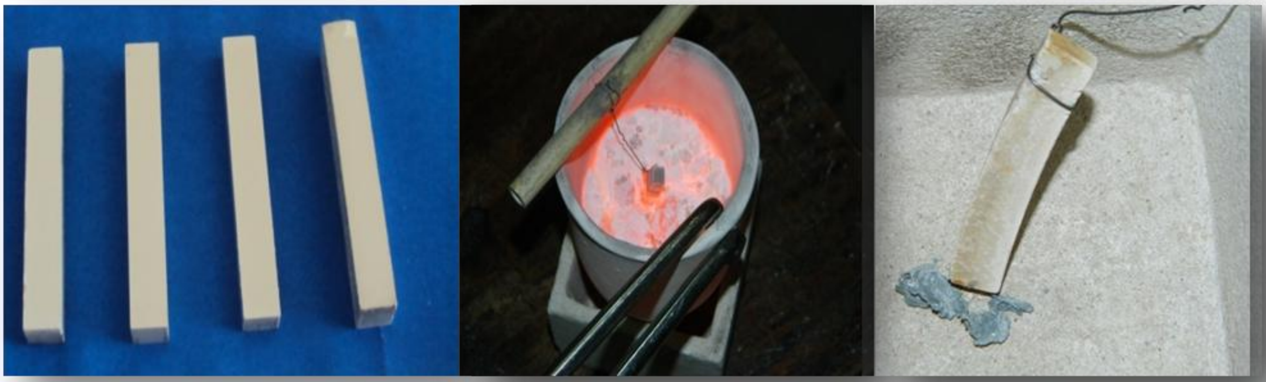

Experimental studies carried out using lanthanum phosphate bars with molten aluminum metal at 800°C indicated no reactivity. On cooling, after immersing the bar in aluminum for about 20 minutes, the chunks of metal could easily be peeled off from the $LaPO_4$ surface. This further indicated the non-reactivity of $LaPO_4$with molten metals.

REFERENCES AND NOTES

1. Salavati-Niasari, M., Hosseinzadeh, G., Davar F. Synthesis of lanthanum hydroxide and lanthanum oxide nanoparticles by sonochemical method. *J. Alloy. Compd.* ***509,*** 4098 (2011).

2. Bochl, P.E. Projector augmented-wave method**.** *Phys. Rev. B* ***50***, 17953 (1994)

3. Kresse, G., Furthmüller, J. Efficient iterative schemes for *ab initio* total-energy calculations using a plane-wave basis set. *Phys. Rev. B 54***,** 11169 (1996).

4. Kresse, G., Joubert, D. From ultrasoft pseudo potentials to the projector augmented-wave method. *Phys. Rev. B* ***58***, 1758 (1999).

5. Perdew, J.P., Burke, K., Ernzerhof, M. Generalized Gradient Approximation Made Simple. *Phys. Rev. Lett.* ***77,*** 3865 (1996).

6. Anisimov, V.I., Zaanen, J., Andersen, O.K. Band theory and Mott insulators: Hubbard *U* instead of Stoner *I. Phys. Rev. B* ***44,*** 943 (1991).

7. Anisimov, V.I., Solovyev, I.V., Korotin, M.A., Czyzyk, M.T., Sawatzky, G. A., Density-functional theory and NiO photoemission spectra. *Phys. Rev. B* ***48***, 16929 (1993).

8. Mullica, D.F., Milligan, W.O., Grossie, D.A., Beall, G.W., Boatner, L.A. Nine fold coordination $LaPO_4$: Pentagonal interpenetrating tetrahedral polyhedron. *Inorg. Chim. Acta* ***95***, 231 (1984).
